# Correlation between salivary immunoglobulin A and interleukin-1beta in smokers with dental caries

**DOI:** 10.12688/f1000research.129649.2

**Published:** 2023-05-05

**Authors:** Jaber Al-ibraheem, Yassir Zyara, Nibrass Al-Quraine, Wasna’a M. Abdulridha

**Affiliations:** 1 Department of Conservative, Faculty of Dentistry, University of Kufa, Najaf, Iraq; 2 Department of Conservative, Faculty of Dentistry, The Islamic University, Najaf, Iraq; 3 Department of Physics, Faculty of Science, University of Kufa, Najaf, Iraq

**Keywords:** dental caries, saliva, immunoglobulin A, interleukin-1beta

## Abstract

**Introduction**. Dental caries is one of the most common infectious diseases of the oral cavity, and is an inflammatory disease caused by several factors. Interleukin-1β (IL-1β) is a major mediator of acute inflammation and essential for the development of specific immune responses. The objective of this study was to assess the levels of secretory IgA (s-IgA) and IL-1β in the saliva of smokers with dental caries, and to discover the correlation between these parameters and dental caries.

**Methods**. Saliva samples were collected from 30 smokers, age range 21–70 years, with dental caries, in addition to 18 healthy non-smoker volunteers, age range 21–65 years. s-IgA and IL-1β levels in the saliva samples were estimated by enzyme-linked immunosorbent assay (ELISA).

**Results**. The mean saliva IgA levels between smokers with dental caries group and healthy subjects were not significantly different (p=0.077), while the saliva levels of IL-1β were higher in the smokers in dental caries group, with a significant difference of p<0.05. No significant associations were found between s-IgA and IL-1β levels, and other parameters such as age, sex, body mass index (BMI) and C-reactive protein (CRP) (p>0.05). There were highly positive associations and significant differences between IL-1β and CRP levels in the two groups under the study (p=0.006).

**Conclusions**. Our study revealed a significant increase in IL-1β levels in saliva of smokers with dental caries, and a positive association between IL-1β levels and caries disease. There is no significant relationship between elevated IL-1β levels and s-IgA in smokers with dental caries.

## Introduction

Dental caries is a type of chronic oral disease that seriously threatens human beings’ health. Although dentists and researchers have struggled for decades with this oral disease, the incidence and prevalence of dental caries is still very high. Therefore, improving disease management and life cycle management of dental caries is a major issue for all populations. Therefore, a clinical difficulty assessment system for the prevention and management of dental caries was developed based on the diagnosis and classification of dental caries. ^1^ It is a widespread infectious disease, caused by several factors determined by the dynamic balance between pathological and protective factors. ^2^^,^^3^ Those agents cause local destruction of sensitive hard tooth tissues by acidic byproducts of bacterial fermentation of dietary carbohydrates. ^4^ Saliva plays an important role in maintaining oral health, helping to build and maintain healthy soft and hard tissues. When saliva flow is reduced, oral health problems such as dental caries and oral infections can develop. ^3^^,^^5^ The protective function of saliva can overwhelm bacterial action, so studies indicate the importance of prevention and treatment, as is the case in other infectious diseases. ^2^ Saliva contains antimicrobial proteins, *i.e.,* immunoglobulin A (IgA), immunoglobulin G (IgG), and immunoglobulin M (IgM), as well as antimicrobial agents, *i.e.,* lysozyme, prolene-rich glycoproteins, mucins, lactoferrin, and lactoperoxidases. These parameters are present in low concentrations, but they work by means of a synergistic effect in reducing metabolism and bacterial growth, in addition to being an effective network for oral defense. ^6^

C-reactive protein (CRP) is a protein made in hepatocytes as a result of interleukin-6 (IL-6) and IL-1 stimulation in the acute phase. It belongs to the pentraxin family and is increased in cases of inflammation, injury or infection. ^7^ Cytokines play a major role in modulating the immune response, and are either pro-inflammatory (tumor necrosis factor alpha (TNF-α), IL-1β, transforming growth factor beta (TGF-β), IL-6, and IL-8), or anti-inflammatory (IL-4, IL-10, IL-12, and interferon gamma (IFN-γ)). The nucleus of any cell has the ability to secrete cytokines, but their main source is macrophages and helper T-cells. ^8^ Among the cytokines, interleukins have a crucial function and are implicated in oral cancer. All markers are also important to development the specific immune responses. ^9^ IL-1β and TNF-α have both been considered essential mediators of acute inflammation. ^5^ Our current study aims to assess the levels of secretory IgA (s-IgA) and IL-1β in smokers with dental caries, knowing the correlation between these parameters and caries disease, as well as knowing the relationship between these two parameters in the same patients.

## Methods

### Study population

The present study consisted of 48 volunteers, age range 21–70 years, divided into two groups. The first group included 18 healthy non-smoker volunteers (11 male and seven female), age range 21–65 years, as controls, while the second group included 30 smokers with dental caries (17 male and 13 female), age range 21–70 years, enrolled at the Clinical Center from College of Dentistry at University of Kufa and Al-Kafeel, between October 2021 and March 2022, and they were diagnosed under Dr. Yasser Zyara supervision. Approval for the study was obtained from the Kufa and Al-Kafeel University Ethics Commission for human subjects. The study protocol was explained to all participants and written informed consent was taken from all before clinical examination or evaluation procedures, *e.g.* examination of teeth, periodontal, oral mucosal status, assessment of malocclusion, and collection of saliva samples. Also, all patients had a clinical periodontal examination. Individuals with medical disorders such as diabetes mellitus, pregnant or nursing females, and those who had antibiotic drugs or periodontal therapy in the previous three months were excluded from the study. The body mass index (BMI) of each volunteer was calculated in a standard manner.

### Saliva sampling

Whole saliva samples were obtained by expectorating into sterile tubes, during which volunteers were requested not to drink (except water) or to chew gum at least one hour before the assembly. Saliva samples were collected between 09:00 and 11:00 AM. The saliva was collected for 10 min in titration tubes by the drooling method. The saliva samples were clarified by centrifugation (800g) for 10 min at 4°C, then immediately aliquoted and stored at −80°C until required for assays. ^10^

### ELISA analysis of IL-1β and s-IgA in saliva

The concentration of IL-1β in saliva was detected by using human Enzyme-Linked Immunosorbent Assay (ELISA) Kits (Elabscience, USA). The ELISA kit uses a sandwich ELISA in accordance with the manufacturer’s instructions. Absorbance in each well was read on an ELISA reader using 450 nm as the primary wavelength. Concentrations of IL-1β in the saliva were estimated using the standard curve. The level of s-IgA in saliva samples was detected by using ELISA kits (Cayman Chemical, Michigan, USA), according to the manufacturer's instructions. CRP levels were measured by using the human CRP ELISA kit (Diaclone, Besancon, FR). To assess the effect of periodontal disease on the salivary cytokine levels, the community periodontal index of treatment needs (CPITN) was measured in each participant after saliva collection. ^11^ Patients with chronic inflammatory diseases, such as psoriasis, arthritis, Sjögren’s syndrome, and inflammatory bowel disease that may affect levels of salivary cytokines were excluded.

### Statistical analysis

All statistical analyses were performed by using the SPSS v.24 software program (RRID: SCR_002865, https://scicrunch.org/) *.* Data were expressed as mean ± SD. The significance of the difference between the mean values of the measured parameters between groups were evaluated by the student’s t-test, and chi-square. Correlation was indicated by the Pearson correlation coefficient test, and p<0.05 was considered significant.

## Results

This study enrolled 30 smokers with dental caries, in addition to 18 healthy non-smoker volunteers. Statistical analysis of the age of the study subjects showed no significant difference between the mean age of the smokers with dental caries group (41.82 ± 12.459) and healthy group (40.68 ± 12.356; p=0.690), as shown in Table 1. The mean BMI, age, and sex between the experimental and control groups were similar and there was no statistically significant differences (p=0.556, p=0.690, p=0.467, respectively). There was a significant difference in CRP between the two groups (p=0.028). No statistically significant difference in periodontal health assessed by the *Community Periodontal Index of Treatment Needs* (CPITN) was observed between the two groups (p=0.511), as shown in Table 1. The mean saliva IgA levels among smokers with dental caries group and healthy subjects were not significantly different (p=0.077), while the saliva levels of IL-1β were high in the smokers with dental caries group with significant differences from the healthy group (p<0.05), as shown in Table 2. No significant associations were found between s-IgA and IL-1β levels; with other parameters such as age, sex, BMI and CRP p>0.05. A highly positive correlation and significant differences between IL-1β and CRP levels were observed in the studied groups (p=0.006), as shown in Table 3.

**Table 1.** Comparison between smokers with dental caries and healthy group, according to BMI, age, sex, CRP, and CPITN.

Parameters	Dental caries cases (n=30) (mean ± SD)	Healthy group (n=18) (mean ± SD)	P-value
BMI (kg/m ^2^)	21.03 ± 7.12	22.11 ± 6.23	0.556
Age (years)	41.82 ± 12.459	40.68 ± 12.356	0.690
Sex	26.44 ± 6.16	27.17 ± 4.38	0.467
CRP (mg/L)	10.67 ± 2.61	3.06 ± 2.35	0.028 ^*^
CPITN	2.31 ± 1.13	2.19 ± 1.08	0.511

* p<0.05: Differences between patients with dental caries and healthy group.

**Table 2.** Salivary IgA and IL-1β in smokers with dental caries and healthy group.

Parameters	Dental caries cases (n=30) (mean ± SD)	Healthy group (n=18) (mean ± SD)	p-value
IgA (mg/dl)	23.12 ± 10.71	20.53 ± 5.36	0.077
IL-1β (pg/mL)	902.11 ± 61.22	350.15 ± 60.33	0.006 ^*^

* p<0.05: Differences between patients with dental caries and healthy group.

**Table 3.** Correlation between salivary IgA, IL-1β, CRP, BMI, age, and sex in *smokers* with dental caries.

Parameters	IgA	IL-1β	CRP	Age	Sex	BMI
IgA (mg/dl)	-	0.778	0.244	0.184	0.287	0.262
IL-1β(pg/mL)	0.778	-	0.006	0.074	0.086	0.088

## Discussion

Dental caries is a diet-related disease that continues to be a problem for some dental patients. Saliva is well formulated to protect against dental caries. Saliva’s buffering capability, the ability of saliva to wash the surface of the tooth, clear bacteria, control purification and mineralization, and saliva’s antibacterial activities and other mechanisms all contribute to its primary role in the health of teeth. ^1^^,^^3^^,^^12^ Several studies have confirmed that knowledge of the functional properties of saliva as well as those of its separate components allows for a better assessment of susceptibility to dental caries. ^12^

Cytokines play a large role in regulating many aspects of the immune response, so they are useful tools for diagnosing and monitoring the oral cavity along with other factors. ^2^^,^^5^ One of the recent studies indicated the possibility of using saliva as a diagnostic material to measure the biomarkers released during the onset and progression of the disease. ^5^ Immune cell products (IL-1β, TNF-α, IL-6) play an important role in oral mucosal pathology, but the exact role of cytokines in the etiology of dental caries is not well known. In innate responses, the molecular phenotypes associated with the oral pathogen bind to host cell receptors, including dendritic cells that have a role in activating the acute inflammatory response by releasing active pro-inflammatory cytokines such as IL-1β, IL-6, and TNF-α. ^7^ On the other hand, in cases where periodontitis develops with potent inflammatory responses, gingivitis will disappear, whereas if infection persists and bacterial proliferation occurs, intensification of the warning response can damage periodontal tissues in the affected patients. ^13^

The results of our study indicated that the mean BMI, age, and sex between the experimental (dental caries) and control (healthy) groups were similar and not statistically significant (p=0.556, p=0.690, p=0.467, respectively), as shown in Table 1. Conversely, significant differences were observed in the CRP levels between the two groups (p=0.028), but there was no statistically significant difference in periodontal health between the two groups (p=0.511) when assessed by CPITN. These results are in agreement with other studies. ^13^^,^^14^ A previous study indicated that high levels of CRP are a risk factor for one type of cancer. Therefore, high levels of CRP before surgery can be considered an undesirable diagnostic indicator. ^15^ Yadav *et al.* ^16^ noted that the pattern of dental caries varies with BMI, sex, age, race, food habits, oral hygiene practices, socio economic status, and geographical location. Also, they indicated a relationship between CRP levels and disease prognosis. These results are consistent with the results of our current study. Park *et al.* ^17^ noted the high CRP/albumin ratio in cases of dental caries and its association with disease for long periods, and thus decreased survival rates. A previous study indicated the possibility of using CRP, haemoglobin, and ferritin as vital markers in diagnosing diseases and knowing their development. ^18^ Tampa *et al.* ^7^ concluded that tumors induce inflammatory responses by increasing CRP levels in serum, and the chronic inflammation contributes to progression of the malignancy. Grimm *et al.* ^19^ were able to identify a large number of total leukocytes, neutrophils, and high levels of CRP, and also they indicated that a small number of lymphocytes is associated with a lower survival rate in oral cancer.

The mean salivary IgA between smokers with dental caries and the healthy subjects group was not significant (p=0.077), while the IL-1β rate was statistically different (p<0.05), see Table 2. These results are confirmed by other studies. ^13^^,^^20^ Several studies showed an increase in s-IgA levels during the caries process compared with caries-free subjects. ^2^^,^^21^ Despite numerous studies, the correlation between s-IgA and dental caries remains unclear. On the other hand, previous studies have shown that elevated salivary IgA levels are associated with decreased dental caries activity, and that s-IgA production provides a local defense with regards to dental infection. ^22^^,^^23^ Brandtzaeg ^24^ suggested that elevated IgA levels are a protective response to reduce dental caries, and also indicated that large numbers of carcinogenic bacteria in untreated necrotic lesions could stimulate an immune response and increase salivary IgA levels in the mouth. Salivary cytokine levels were elevated due to disorders of the oral cavity ( Table 2), and these results were consistent with previous studies. ^13^^,^^14^ In our study, the mean salivary IgA rate was not significant, while the IL-1β rate was statistically different between the dental caries group and healthy subjects. Gomes *et al.* ^25^ noted that salivary IL-1β and TNF-α levels increase in periodontal disease and are associated with the onset and increased severity of the disease, and that their levels decrease after treatment. Also, they showed that IL-1β and TNF-α are promising biomarkers in detecting periodontal disease. IL-1β plays an important role in the acute inflammatory response, and persists for a relatively long time at the site of inflammation. ^7^ Cogulu *et al.* ^9^ observed a significant increase in levels of CRP and IL-6 in saliva of the patients diagnosed with apical periodontitis compared with people with normal or healthy teeth. Gomes *et al.* ^25^ noted the presence of inflammatory cytokines represented by TNF-α and IL-1β in saliva of patients in the early stages, and they indicated their important role in the development of periodontal disease; they concluded that elevated levels of inflammatory cytokines are evidence of disease progression. Several reports indicated the role of acute chronic inflammation in the occurrence and development of carcinogenesis through the modulation of inflammatory cells, and cytokine production, ^7^^,^^26^ so it can explain why levels of some cytokines including IL-1β are elevated in patients. These studies support the results of our current study.

The IL-1β receptor stimulates the activation of signal transducers, Janus kinases (JAK), and activators of transcription (STATs), which then stimulate pathways involving mitogen-activated protein kinase (MAPK), which in turn supports the development of carcinogenesis, and this may explain the reasons for the elevated IL-1β levels in the saliva of the smokers diagnosed with dental caries. The results of this study were validated by ^27^ where they found that higher pro-inflammatory cytokines such as IL-1β in dental caries patients are responsible for the lower number of stony cells and osteoblasts; they also indicated that demineralization of the teeth and the development of cavities occurs in a high incidence in smokers. On the other hand, a tumor can trigger an inflammatory response, causing the release of cytokines *i.e.,* IL-1β and IL-6. ^28^ Gornowicz *et al.* ^2^ reported increased IL-1β levels in the healthy periodontal group compared with the chronic periodontitis group. Colak *et al.* ^29^ showed that infants frequently exposed to breast milk, formula milk, sugary liquids, fruit juices and other sweet-tasting liquids for prolonged periods are at risk of early childhood caries (ECC). In contrast to our results, ^27^ noted increased pro-inflammatory cytokines levels in the saliva of smokers with dental caries results in fewer broblasts and osteoblasts. Mclachlan *et al.* ^30^ concluded that the concentrations of pro-inflammatory cytokine are also increased in both gingival tissues and serum of people with periodontal inflammation. Our study indicated that there was no significant correlation between s-IgA and IL-1β levels, and with other parameters such as age, sex, and BMI p>0.05, but there was a positive association between IL-1β levels and CRP (p=0.006, Table 3).

The results of our current study are consistent with previous studies. ^2^^,^^13^^,^^20^ Significant changes in IL-1β levels provide us with great potential for their use as effective biomarkers in saliva. This could open new avenues for treatment plans of targeted leukotriene therapy. ^30^ This conclusion was confirmed by previous reports. ^5^^,^^13^ Contrasting with the results of our study, ^31^ reported that dental caries was not significantly associated with IL-1β, *interleukin-1 receptor antagonist* IL-1Ra), or IL-10 concentrations in the serum and saliva of patients. On the other hand, several studies confirmed that the high level of salivary cytokines occurs as a result of disorders in the oral cavity, ^2^^,^^5^^,^^32^ and this conclusion is consistent with our current results. However, these results are not consistent with previous studies. ^5^^,^^33^^,^^34^ On the other hand, ^35^ indicated a significant reversible association between s-IgA and acute activity. A positive association was observed between an elevated IL-1β level and dental caries, while there was no positive association between increased levels of IgA and dental caries. These results are consistent with research highlighting that there is no association between dental caries and increased levels of IgA. ^13^^,^^36^

Future research is necessary to fully characterize the salivary components and their interactions and how these affect the dental caries process. With this knowledge, the use of modified oral molecules as therapeutic agents may become a reality. Also of interest is the potential to influence the secretion of salivary components through increased knowledge and control of the secretory processes responsible for the delivery of those components. The results of our current study suggest a link between the production of inflammatory and immunoregulatory cytokines represented by IL-1β in smokers with dental caries, so this parameter can be used as an important biomarker to indicate the disease severity and progression. Finally, when developing a treatment plan based on the assessment of caries risk and activity of carious lesions, we need to develop a patient-centered and personalized treatment plan to restore the delicate environmental balance of the oral cavity, to control the progression of caries and to restore the structure, and function of the carious teeth.

## Conclusions

In smokers, a positive association was found between dental caries and elevated IL-1β levels, while no significant differences were found in the level of S-IgA and caries. The present study also indicated no significant correlation between elevated IL-1β levels and s-IgA in smokers with caries disease.

## Ethical approval

Approval for the study was obtained from the Kufa and Al-Kafeel University Ethics Commission for human subjects. Ethical approval committee no. 1730/2022, Kufa University, Iraq

## Consent

The study protocol was explained to all participants, and written informed consent was taken from all before clinical examination or evaluation procedures.

## Data Availability

Figshare. Correlation between Salivary Immuno Globulin A and Interleukin-1beta in Smokers with dental caries. https://doi.org/10.6084/m9.figshare.21909753.v1. ^37^ This project contains the following underlying data:
Raw data for comparison between smokers with dental caries and healthy group, according to BMI, age, sex, CRP, and CPITN Data are available under the terms of the Creative Commons Attribution 4.0 International license (CC-BY 4.0).
