# Supplementary material for: Correlation between salivary immunoglobulin A and interleukin-1beta in smokers with dental caries
Source: F1000Res. 2023 May 5;12:175. Originally published 2023 Feb 14. [Version 2] doi: 10.12688/f1000research.129649.2 (PMC10173202; doi:10.12688/f1000research.129649.2)
Supplement: Peer review history

# Peer review: Reviewer response for version 2

**DOI:** 10.5256/f1000research.146487.r172052

**Published:** 2023-05-09

**Authors:** Neihaya Heikmat Zaki

**Affiliations:** 1 Department of Biology, College of Science, Mustansiriyah University, Baghdad, Iraq

No further comments to make.

Is the work clearly and accurately presented and does it cite the current literature?

Yes

If applicable, is the statistical analysis and its interpretation appropriate?

Yes

Are all the source data underlying the results available to ensure full reproducibility?

Yes

Is the study design appropriate and is the work technically sound?

Yes

Are the conclusions drawn adequately supported by the results?

Yes

Are sufficient details of methods and analysis provided to allow replication by others?

Yes

Reviewer Expertise:

Biotechnology

I confirm that I have read this submission and believe that I have an appropriate level of expertise to confirm that it is of an acceptable scientific standard.

# Peer review: Reviewer response for version 1

**DOI:** 10.5256/f1000research.142347.r163659

**Published:** 2023-03-27

**Authors:** Ghizal Fatima

**Affiliations:** 1 Department of Biotechnology, Era's Lucknow Medical College and Hospital, Lucknow, Uttar Pradesh, India

Authors tried to explain and assess the levels of secretory IgA (s-IgA) and IL-1β in the saliva of smokers with dental caries, and they reached to a conclusion that there is no significant relationship between elevated IL-1β levels and s-IgA in smokers with dental caries. The whole paper is written in a well presented manner.

 It is an important topic, especially taking important secretary markers like IgA (s-IgA) and IL-1β in the saliva of smokers with dental caries, and to discover the correlation between these parameters and dental caries. Despite a lot of substantial work has been done in this area, this work will make important contribution in the field of dental caries, as they have taken different markers for assessment.

 There are just little or no issues with the manuscript that needs to be clarified/addressed. This article is nicely written, it addresses all the scientific questions and answers in the paper, and the statistical analysis is also nicely done. Figures and tables are synchronized in a well manner presentation. Furthermore, the discussion and conclusion parts arewell written, while results are given in an elaborated manner just as they warrant.

 Below are few specific comments by section:

**Abstract:** This section needs to be rechecked again for grammar.

**Introduction:** More information about the correlation in secretary markers with the dental caries has to be explained in detail.

**Methods:** This section is well written; it explained all the methods done in the study in a detailed manner.

**References: **All the recent references are taken in the study.

Is the work clearly and accurately presented and does it cite the current literature?

Yes

If applicable, is the statistical analysis and its interpretation appropriate?

Yes

Are all the source data underlying the results available to ensure full reproducibility?

Yes

Is the study design appropriate and is the work technically sound?

Yes

Are the conclusions drawn adequately supported by the results?

Yes

Are sufficient details of methods and analysis provided to allow replication by others?

Yes

Reviewer Expertise:

Biomedical sciences

I confirm that I have read this submission and believe that I have an appropriate level of expertise to confirm that it is of an acceptable scientific standard.

# Peer review: Reviewer response for version 1

**DOI:** 10.5256/f1000research.142347.r163658

**Published:** 2023-02-21

**Authors:** Neihaya Heikmat Zaki

**Affiliations:** 1 Department of Biology, College of Science, Mustansiriyah University, Baghdad, Iraq

A brief summary of the article, "Correlation between salivary immunoglobulin A and interleukin-1beta in smokers with dental caries":

 There were highly positive associations and significant differences between IL-1β and CRP levels in the two groups under the study. While no significant differences were found in the level of S-IgA and caries. This study also indicated no significant correlation between elevated IL-1β levels and s-IgA in smokers with caries disease.

 There are some questions that must be answered by authors:

1. Why not include CRP in title and keywords because this parameter has significant differences, as mentioned in results?
2. There is a difference in numbers and age range of volunteers between the two groups under study, as mentioned in methods (page 3).
3. In the study population, you must mention the doctor who supervised the excluded patients with disorders and disease.

Comments can also be found here.

Is the work clearly and accurately presented and does it cite the current literature?

Yes

If applicable, is the statistical analysis and its interpretation appropriate?

Yes

Are all the source data underlying the results available to ensure full reproducibility?

Yes

Is the study design appropriate and is the work technically sound?

Yes

Are the conclusions drawn adequately supported by the results?

Yes

Are sufficient details of methods and analysis provided to allow replication by others?

Yes

Reviewer Expertise:

Biotechnology

I confirm that I have read this submission and believe that I have an appropriate level of expertise to confirm that it is of an acceptable scientific standard, however I have significant reservations, as outlined above.
